# Investigation on Fatigue Performance of Diatomite/Basalt Fiber Composite Modified Asphalt Mixture

**DOI:** 10.3390/polym14030414

**Published:** 2022-01-20

**Authors:** Chunfeng Zhu, Huijin Luo, Wei Tian, Binbin Teng, Yongmei Qian, Huaxue Ai, Bo Xiao

**Affiliations:** 1 College of Civil Engineering, Jilin Jianzhu University, Changchun 130118, China; zhuchunfeng@jlju.edu.cn (C.Z.); luohuijin@jlju.edu.cn (H.L.); tengbinbin@jlju.edu.cn (B.T.); qianyongmei@jlju.edu.cn (Y.Q.); aihuaxue@jlju.edu.cn (H.A.); 2 Changchun Municipal Engineering Design & Research Institute, Changchun 130033, China; xiaobo_1980@sina.com

**Keywords:** fatigue performance, diatomite, basalt fiber, asphalt mixture, four-point bending fatigue test, grey correlation analysis

## Abstract

The fatigue resistance of asphalt mixture is an important indicator to evaluate the durability of asphalt pavement. In order to improve the fatigue properties of asphalt mixture, diatomite and environmental basalt fiber were added. Four types of asphalt mixtures, ordinary asphalt mixture (AM), diatomite modified asphalt mixture (DAM), basalt fiber modified asphalt mixture (BFAM) and diatomite/basalt fiber composite modified asphalt mixture (DBFAM), were chosen, whose optimum asphalt–aggregate ratio, optimum content of diatomite and optimum content of basalt fiber could be determined by Marshall test and response surface methodology (RSM). The multi-functional pneumatic servo Cooper test machine was carried out by a four-point bending fatigue test. Through the comparative analysis of flexural-tensile stiffness modulus (*S*), initial stiffness modulus(*S*_0_), residual stiffness modulus ratio, lag angle (*ϕ*) and cumulative dissipation energy (*E_CD_*), the fatigue resistance of asphalt mixture can be effectively improved by adding diatomite and basalt fiber. Grey correlation analysis was also used to analyze the degree of correlation between the fatigue life and the influencing factors such as VV, VMA, VFA, OAC, *S*, and *E_CD_*. The analysis results indicate that *E_CD_* has the greatest impact on the fatigue life of the asphalt mixture.

## 1. Introduction

Asphalt is a mixture of black-brown polymers composed of hydrocarbons with different molecular weights and their non-metallic derivatives. Asphalt is mainly used in coatings, plastics, rubber and other industries and paving pavements. Asphalt is a widely used pavement structural cementitious material in road engineering. It can be mixed with different mineral materials in proportion to build asphalt pavement with different structures. Improving the performance of asphalt or asphalt mixture is an important means to prolong the service life of pavements. In recent years, many researchers have improved the performance of asphalt or asphalt mixture by adding modified substances. The anti-fatigue characteristic of the asphalt mixture is one of the important indicators of road performance.

Fatigue refers to a phenomenon caused by the accumulation of unrecoverable strength attenuation of asphalt mixture pavement under repeated loads [1,2,3]. In the service process, asphalt pavement is subjected to the repeated action of vehicle load and temperature stress for a long time. With the increase of load times, defects and microcracks will occur in the asphalt mixture [4,5]. Moreover, these defects and micro-cracks will continue to extend under dynamic load, the strength of the pavement structure will gradually decay and finally, fatigue failure and cracks will occur in the pavement. In order to ensure that the asphalt pavement has good usability and durability, the asphalt mixture must have good fatigue resistance. Fatigue life is an important indicator to evaluate the service life of asphalt pavement [6,7,8].

In order to improve the fatigue of asphalt pavement, many researchers add different types of reinforcement materials in asphalt mixtures, such as cellulose, lignin, glass fiber, polyester fiber, inorganic fiber and so on [9,10,11,12,13]. Zheng et al. [14] studied the fatigue property of basalt fiber-modified asphalt mixtures under complicated environments. Zhao et al. [15] investigated the effect of basalt fiber on the road performance of asphalt mixture. Riekstins et al. [16] evaluated the anti-fatigue property of brucite fiber reinforced asphalt mixture under sulfate and dry-wet circle corrosion environments. Lou et al. [17] evaluated the enhancement effect of basalt fiber on the fatigue performance of the mixtures.

Considering the climatic characteristics of the seasonal freezing region in Jilin Province, China, this study mixed diatomite and basalt fiber into asphalt mixtures and evaluated their improvement effect on the fatigue resistance of asphalt mixture through fatigue test. A fatigue test mainly employs phenomenon mechanics and mechanics approximation methods [18,19], but there are more experimental methods for using phenomenon mechanics at present. Due to the low cost and short cycle of laboratory tests, they are widely used. An indoor fatigue test mainly includes a repeated bending test, support bending test, uniaxial test, triaxial test, indirect tensile test and so on. SHRPA-003A indicates that the repeated bending fatigue test (especially the four-point bending test) is most conforming to the actual pavement stress, and it is more reasonable to use the four-point bending fatigue test of the rectangular beam as the standard test for the fatigue performance of asphalt mixture. Yu et al. [20,21] studied three asphalt mixture fatigue testing machines, James Cox & Sons (USA), Cooper µµ(UK) and IPC (Australia). The results showed that the test results of the above three four-point bending fatigue testing machines were consistent. Cooper and IPC fatigue testing machines can obtain lag angle and dissipated energy, which is beneficial to fatigue test research and analysis. Di Benedetto, Cardona DR, Sébastien Lamothe, Zhiyong Wu, Yang Panpan and other scholars studied the fatigue performance of asphalt mixture using dissipated energy, lag angle, cumulative dissipation energy and the ratio of dissipated energy change methods [22,23,24,25,26,27,28].

After careful consideration, this paper uses the multi-functional pneumatic servo Cooper test machine (NU-14) produced by Cooper Research Technology Limited in the UK to carry out a four-point bending fatigue test, testing the initial stiffness modulus, stress value, strain value, lag angle, cumulative dissipation energy and fatigue life, etc. The factors affecting fatigue life are analyzed by grey correlation theory.

## 2. Experiments and Methods

### 2.1. Raw Materials

**Figure 1:**
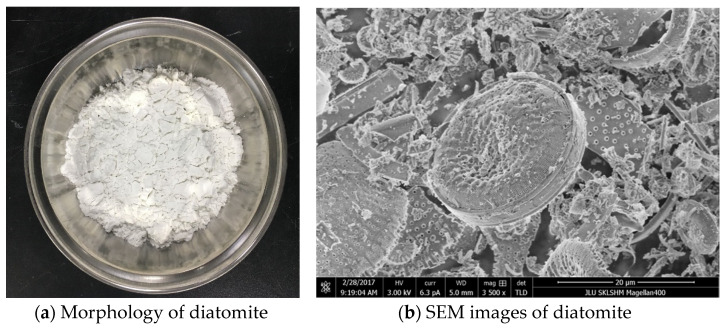
Diatomite.

**Figure 2:**
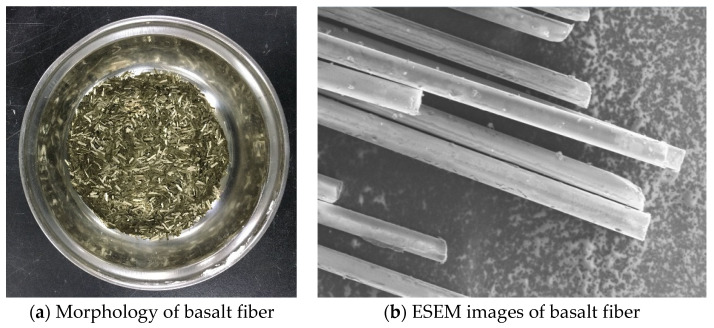
Basalt fiber.

The asphalt AH-90 in this paper was supplied by Panjin Petrochemical Industry, located in Liaoning Province of China. The physical properties of the asphalt were measured according to Chinese specification JTG E20-2011 [29]; the measured results are presented in Table 1. The ecofriendly diatomite was obtained from Changbai Mountain in Jilin Province. The average size of diatomite particles is approximately 10 µm and its shape is a disc under the microscope, as shown in Figure 1. The basalt fiber was supplied from Jiuxin Basalt Industry Co., Ltd. in Jilin Province, as shown in Figure 2, and the physical properties of these materials are presented in Table 2, Table 3, Table 4 and Table 5 [30]. The laboratory temperature was 20 ± 2 °C; the humidity was 60%.

The used aggregates were basalt produced in Liaoyuan, Jilin Province. The properties of aggregates were tested in accordance with the Chinese specification JTG E42-2005 [31]. The technical properties of aggregates are listed in Table 6 and Table 7, which meet the requirements of the Chinese specification JTG F40-2004 [32].

### 2.2. Gradation Design of Asphalt Mixture

**Figure 3:**
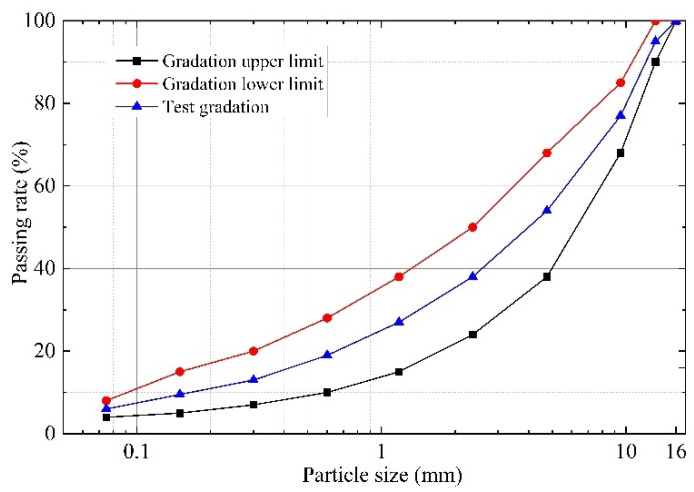
Aggregate gradation curve of AC-13 in this study.

The gradation type of asphalt mixture used in this study was AC-13, and the selected gradation curve is shown in Figure 3 according to Chinese specification JTG F40-2004 [32].

According to the compaction method in the Chinese specification JTG E20-2011 [29], the asphalt mixture was developed into a standard Marshall test piece. The optimum asphalt–aggregate ratio (OAC) of the asphalt mixture is 4.78%, determined by testing the test indexes including VV, VMA, VFA, MS and FL. On this basis, the range of optimum asphalt content of diatomite/basalt fiber composite modified asphalt mixture (DBFAM) could be selected. According to a large number of research data [14,33,34,35,36], it is determined that the content range of basalt fiber is 0.2% to 0.4% and the dosage range of diatomite instead of mineral powder is 5% to 10%. Using the central composite design (CCD) of response surface methodology (RSM) to determine the optimal proportion of the DBFAM [9], the optimum asphalt–aggregate ratio is 5.22%, the optimal content of diatomite is 6.3% and basalt fiber is 0.25%.

In order to ensure the test data are comparable, basalt fiber and diatomite were separately added into AC-13, and the optimum asphalt–aggregate ratios of basalt fiber modified asphalt mixture (BFAM) and diatomite modified asphalt mixture (DAM) were determined to be 5.09% and 5.12%, respectively, based on the Chinese specification JTG F40-2004 [32]. The physical properties of asphalt mixtures are shown in Table 8. The laboratory temperature was 25 ± 2 °C and the humidity was 60% when the test was carried out.

### 2.3. Four-Point Bending Fatigue Test

#### 2.3.1. Preparation of Asphalt Mixture Beam

**Figure 4:**
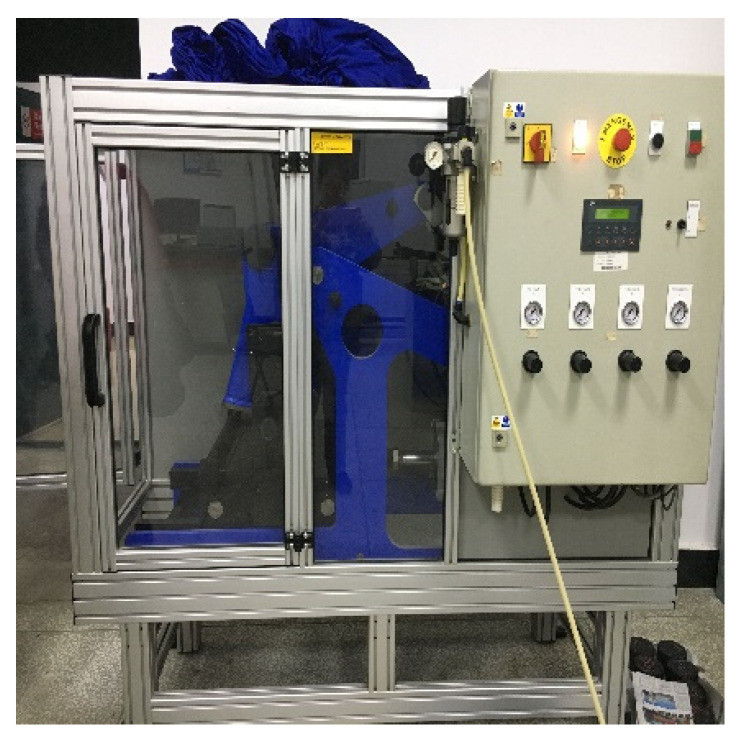
Vibratory roller compacting machine.

The preparation process of the four-point bending fatigue trabecular specimens mainly includes mixture mixing, specimen rolling and forming and trabecular cutting. The mixture mixing was carried out in accordance with the standard Marshall mixture specimen preparation process. The evenly mixed mixture was loaded into the test mold (400 mm × 300 mm × 75 mm), and then formed by high pressure vibration through the asphalt mixture vibratory roller compacting machine produced in Britain. The specimen was formed by controlling the height of the wheel grinding plate, and the air pump pressure was set to 2 bar, with two plates for each component type. The laboratory temperature was 20 ± 2 °C. The molding test equipment is shown in Figure 4.

After the test specimen was formed for 24 h, the formwork was removed, the test plate was cut and the infrared control cutting machine was used. The slab was cut into small beams with dimensions of 380 mm long, 65 mm wide and 50 mm high. Each slab can be cut into four trabecular test beams. In order to ensure that the trabecular is dry enough, the fatigue test was carried out after the test beams were cut for 24 h.

#### 2.3.2. Four-Point Bending Fatigue Test

**Figure 5:**
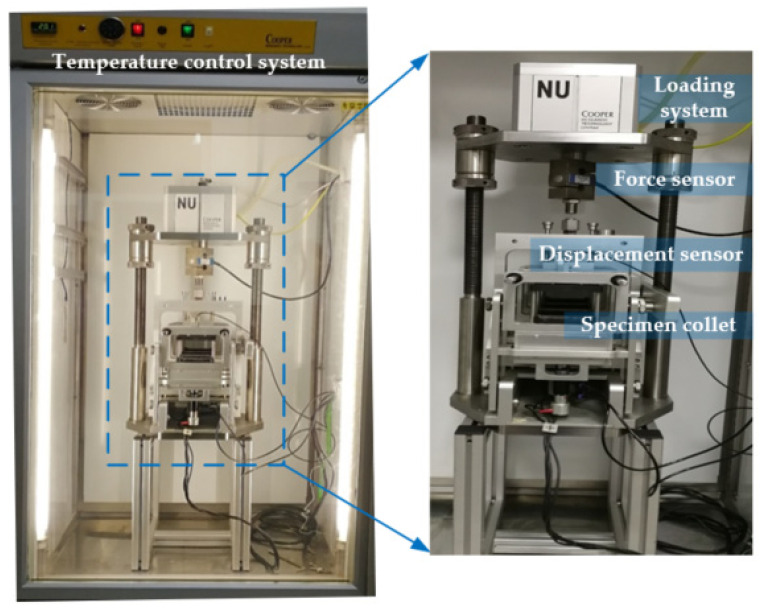
Fatigue test device for four-point bending beam.

The fatigue test was carried out using the Cooper testing machine, and 800 strain level was adopted during loading. The test device is shown in Figure 5.

The test waveform was a sine wave, the frequency was 10 Hz, the test temperature was 20 °C, the relative humidity was 60%, the test failure criterion was that the stiffness modulus of the specimen decreased to 50% of its initial modulus during loading and the cyclic loading times at this time were used as the fatigue life of the asphalt mixture to evaluate the durability of the asphalt material under the action of alternating loads.

The stiffness modulus of the 100th loading cycle was set as the initial stiffness modulus of the test specimen. Before the fatigue test, a vernier caliper was used to accurately measure the length, width and height of the beam, and the data were input into the computer software system (the distance between four points remains the default value:118 mm), setting the corresponding parameters in the software system. Before the test, the specimen was put into the test temperature for at least 4 h, then the beam was installed into the fatigue loading control system, clamped and fixed, and the test parameters were set according to the software interface. The four-point bending fatigue test would obtain the following test indexes: initial stiffness modulus, stiffness modulus attenuation percentage, stress value, strain value, lag angle, cumulative dissipation energy and fatigue life (number of cyclic loads).

#### 2.3.3. Calculation Procedure of Fatigue Performance

- The calculation equation of maximum tensile stress is listed in Equation (1):
  (1) $$\sigma_{t}=\frac{L\times P}{\omega \times h^{2}},$$
  where *σ_t_* is the maximum tensile stress (Pa); *L* is the beam span (the distance between two clamps at the outer end); *P* is a peak load (N); *ω* is beam width (m); *h* the beam height (m).
- The maximum tensile strain can be calculated by Equation (2):
  (2) $$\varepsilon_{t}=\frac{12\times \delta \times h}{3\times L^{2}-4\times a^{2}}$$
  where *ε_t_* is the maximum tensile strain (m/m); *δ* is the maximum strain of beam center (m); *a* is the spacing between adjacent collets (i.e., *L*/3) (m).
- The flexural-tensile stiffness modulus is obtained by Equation (3):
  (3) $$S=\frac{\sigma_{t}}{s_{t}},$$
  where *S* is the flexural-tensile stiffness modulus (Pa).
- The lag angle is calculated according to Equation (4):
  (4) ϕ=360×f×t,
  where *ϕ* is the lag angle (°); *f* is the loading frequency (Hz); *t* is the lag time of strain peak to stress peak (s).
- The single-cycle dissipation energy is calculated by Equation (5):
  (5) $$E_{D}=\pi \times \sigma_{t}\times \varepsilon_{t}\times \sin\phi ,$$
  where *E_D_* is the dissipated energy of a single cycle (J/m^3^).
- The cumulative dissipation energy is calculated according to Equation (6):
  (6) $$E_{CD}=\sum_{i=1}^{n}E_{Di},$$
  where *E_CD_* is the accumulated dissipated energy (J/m^3^); *E_Di_* is the dissipated energy of a single cycle for the *i*-th loading.
- The ratio of dissipated energy change (*RDEC*) is calculated according to Equation (7):
  (7) $$RDEC=\frac{E_{Di}-E_{Di}}{E_{Di}\left(j-i\right)},$$
  where *RDEC* is the ratio of dissipated energy change; *E_Di_* and *E_Dj_* are the dissipated energy when the loading cycles are *i* and *j*, respectively.

### 2.4. Grey Correlation Analysis

Grey system theory puts forward a new analysis method, called the systematic correlation degree analysis method, which measures the degree of correlation between factors according to the degree of similarity or dissimilarity. Because the correlation analysis method is analyzed according to the development trend and can avoid the interaction of various factors, there is no excessive requirement on the sample size, and no typical distribution law is required. The amount of calculation is small, and the quantitative results of the correlation degree will not be inconsistent with the qualitative analysis. This method can analyze the influence of various factors on the fatigue life of asphalt mixture. Relevance is essentially the difference in geometry between curves. The closer the geometry is, the closer the development trend is, and the greater the degree of relevance is [37,38].

The fatigue performance of asphalt mixture is affected by a variety of factors, among which the main factors are s stiffness modulus, asphalt type, asphalt content, porosity, mineral type, gradation type and test conditions. The research shows that in the fatigue test under strain control, the lower the stiffness modulus of the mixture, the smaller the stress required to maintain the same strain, and the crack propagation will continue for a long time. The smaller the stiffness modulus, the longer the fatigue life of the material. Asphalt species, asphalt content, etc., also affect the fatigue life of the asphalt mixture by affecting the stiffness modulus. Porosity, mineral aggregate type and gradation will affect the fatigue life of asphalt mixture by affecting the composition, structure and internal defects of asphalt mixture. In this paper, grey correlation analysis is used to analyze the correlation degree between the relevant influencing factors and the fatigue life of the asphalt mixture.

The reference sequence and comparison sequence should be specified before correlation analysis. In this paper, the fatigue life of the asphalt mixture is taken as a reference sequence, and the factors influencing the fatigue life asphalt mixture are taken as a comparison sequence. The reference number is listed as *x*_0_, *x*_0_ = (*x*_0_ (1), *x*_0_ (2), …, *x*_0_ (*n*)), the comparison is listed as *x*_1_, *x*_1_ = (*x*_1_ (1), *x*_1_ (2), …, *x*_1_ (*n*)).

The correlation coefficient between the comparison curve and the reference curve at time *k* is:

(8) $$\xi_{i}(k)=\frac{\underset{i}{\min}\underset{k}{\min}\left|x_{0}(k)-x_{i}(k)\right|+0.5\underset{i}{\max}\underset{k}{\max}\left|x_{0}(k)-x_{i}(k)\right|}{\left|x_{0}(k)-x_{i}(k)\right|+0.5\underset{i}{\max}\underset{k}{\max}\left|x_{0}(k)-x_{i}(k)\right|},$$

where *ξ_i_*(*k*) is the relative difference between the comparison curve and the reference curve at the kth moment, and this form of relative difference is the correlation coefficient of *x_i_* to *x*_0_ at time *k*. $\underset{i}{\min}\underset{k}{\min}\left|x_{0}(k)-x_{i}(k)\right|$ is the minimum difference between two hierarchies; $\underset{i}{\max}\underset{k}{\max}\left|x_{0}(k)-x_{i}(k)\right|$ is the maximum difference between the two levels.

The general expression of grey correlation degree is expressed in Equation (9):

(9) $$r_{i}=\frac{1}{N}\sum_{i=1}^{N}\xi_{i}\left(k\right),$$

where *r_i_* is the correlation degree between the curve and the reference curve.

## 3. Results and Discussion

### 3.1. Variation Law of Flexural-Tensile Stiffness Modulus

**Figure 6:**
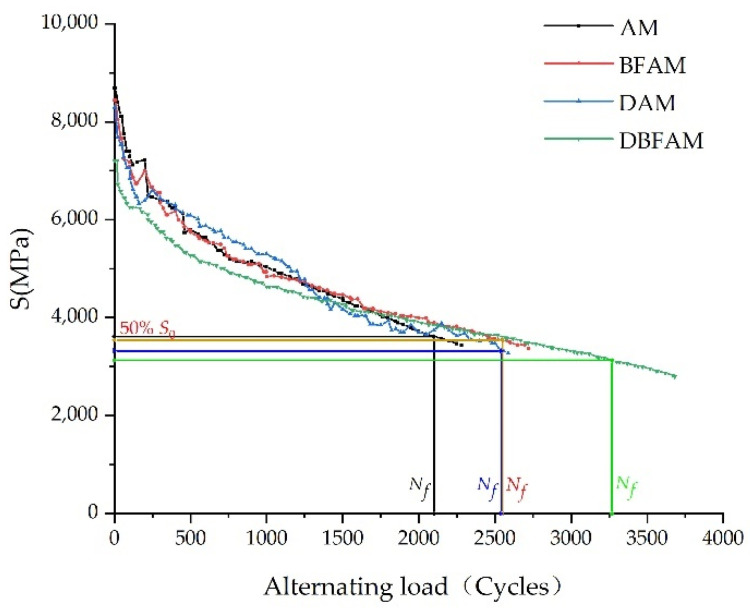
Attenuation law of flexural-tensile stiffness modulus of asphalt mixtures.

The variation trend of flexural tensile stiffness modulus (*S*) of four types of asphalt mixtures with cyclic loading times is shown in Figure 6. It is evident from Figure 6 that the fatigue process of the asphalt mixture mainly goes through two stages before 50% of the initial stiffness modulus. In the first stage, *S* decreased sharply, and in the second stage, *S* decreased slowly and close to linearity. The sharp decrease of S in the first stage is mainly caused by the reorganization of the internal structural materials of the test specimen under the action of alternating load. The second stage is the main stage of material fatigue damage. The test specimen produces fatigue damage under the action of alternating load, and this stage is the initiation and development process of microcracks [39].

The fatigue test results indicated that the fatigue life order of four types of asphalt mixtures is: DBFAM > BFAM ≈ DAM > AM. The addition of diatomite and basalt fiber can significantly improve the fatigue performance of asphalt mixture, and the improvement effect of 0.25% basalt fiber and 6.3% diatomite on the fatigue performance of asphalt mixture is similar. The fatigue life of the asphalt mixture can be greatly improved by adding diatomite and basalt fiber. The test results show that the fatigue life of DBFAM is 55.7% higher than that of AM.

### 3.2. Analysis of Initial Stiffness Modulus

**Figure 7:**
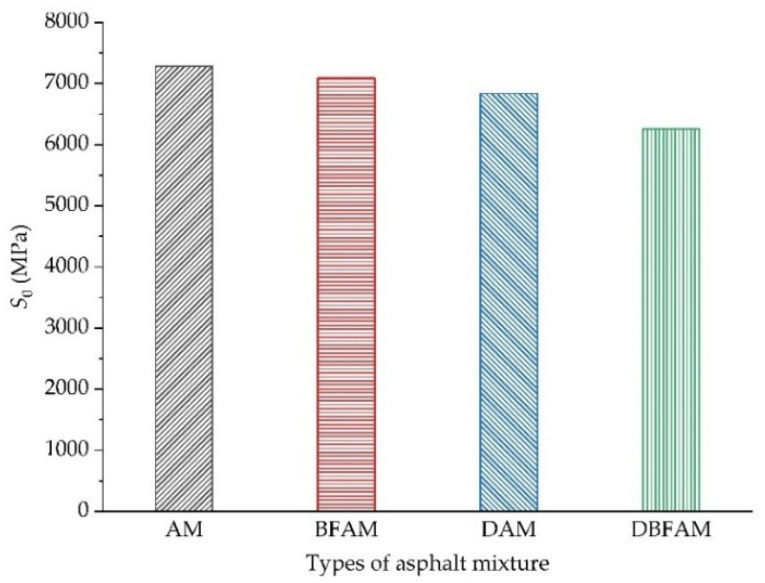
Initial flexural-tensile stiffness modulus test results of asphalt mixture.

The initial flexural tensile stiffness modulus (*S*_0_) is closely related to the determination of the fatigue life of asphalt mixture specimens. In this paper, the stiffness modulus of the 100th loading cycle is uniformly set as the initial stiffness modulus of the test specimens [40]. The experimental results of the initial flexural tensile stiffness modulus of four types of asphalt mixtures are shown in Figure 7. It can be seen from the figure that under the action of 800 strain level, the order of the initial stiffness modulus is AM > BFAM > DAM > DBFAM. The addition of basalt fiber and diatomite increases the optimum bitumen–aggregate ratio of asphalt mixture, and the increase of asphalt content is the main reason for the decrease of initial flexural tensile stiffness modulus.

### 3.3. Analysis of Residual Stiffness Modulus Ratio

**Figure 8:**
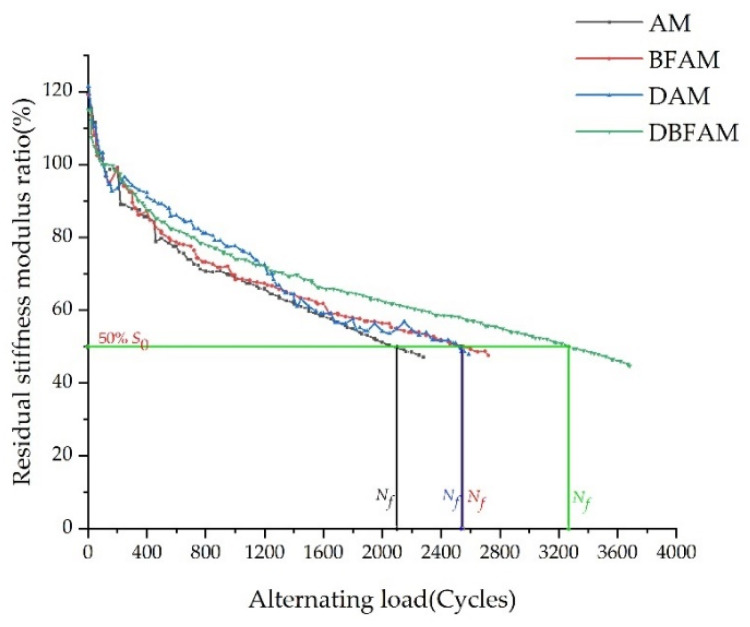
Residual flexural-tensile stiffness modulus ratio of asphalt mixture.

The residual stiffness modulus ratio is defined as the percentage of flexural tensile stiffness modulus corresponding to each alternating load in the initial flexural tensile stiffness modulus, that is, the attenuation rate of flexural tensile stiffness modulus. The relationship between the attenuation rate of flexural-tensile stiffness modulus and fatigue life is shown in Figure 8.

It can be seen from the test results in Figure 7 that the variation trend of the flexural tensile stiffness modulus attenuation rate of four types of asphalt mixtures under alternating load is the same as that of the above flexural tensile stiffness modulus, which is divided into two stages before 50% of the initial stiffness modulus. The first stage is a steep decline, and the second stage drops slowly and nearly linearly.

### 3.4. Analysis of Lag Angle

The lag angle (*ϕ*) is the phase difference between the strain rotation vector and the stress rotation vector [41]. The lag angle (*ϕ*) can reflect the viscoelasticity of the asphalt mixture. The larger *ϕ* is, the more viscous the mixture tends to be. Otherwise, the mixture tends to be elastic [42]. For an ideal elastomer, the lag angle *ϕ* is 0, for purely viscous bodies, *ϕ* is π/2, for viscoelastic materials, *ϕ* is between 0 and π/2 [43].

**Figure 9:**
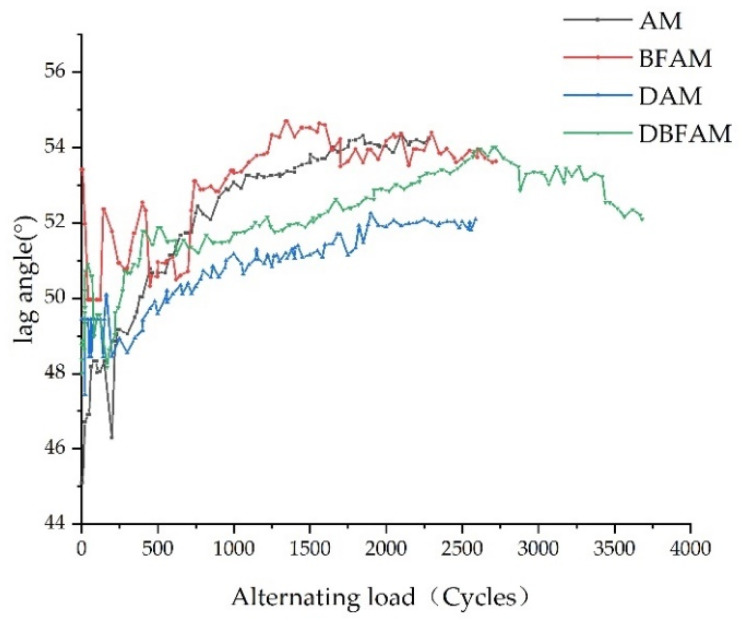
Variation of lag angle of asphalt mixture.

The variation trend of lag angle with loading times is shown in Figure 9. It can be seen from Figure 9 that the lag angle of four types of asphalt mixtures is increasing, but the increase is relatively slow. It shows that the viscoelastic properties of the four types of asphalt mixtures are constantly changing in during the process of the fatigue test. With the increase of loading times, the asphalt mixture continues to develop in the direction of viscosity. This is because, in the process of the fatigue test, the alternating load continuously works on the specimen, resulting in a certain increase in the temperature of the test piece, the lag angle gradually increases, and the asphalt binder changes from elasticity to viscosity. It can be seen from Figure 9 that after the mineral powder is replaced by diatomite, the lag angle becomes smaller and the elasticity of the asphalt mixture increases. The influence of basalt fiber on the lag angle is not very obvious; the viscoelastic change of asphalt mixture is small. Adding diatomite and basalt fiber increases the elasticity of the asphalt mixture. In addition, the fatigue tests of the four kinds of asphalt mixtures are all carried out under the action of high strain level and the time when the materials reach fatigue failure is short. The number of cycles is few, leading to the trend of a slow increase of lag angle.

### 3.5. Analysis of Dissipation Energy

1. Dissipation energy under each load

Asphalt mixture is a type of viscoelastic material. Under the action of alternating load, the stress–strain curves during loading and unloading will not coincide, resulting in a hysteretic curve. The dissipated energy can be determined by the area of this stress–strain hysteretic line and the calculation equation is shown in Equation (5). The variation law of dissipated energy can indirectly reflect the fatigue damage evolution process of asphalt mixture under alternating load.

**Figure 10:**
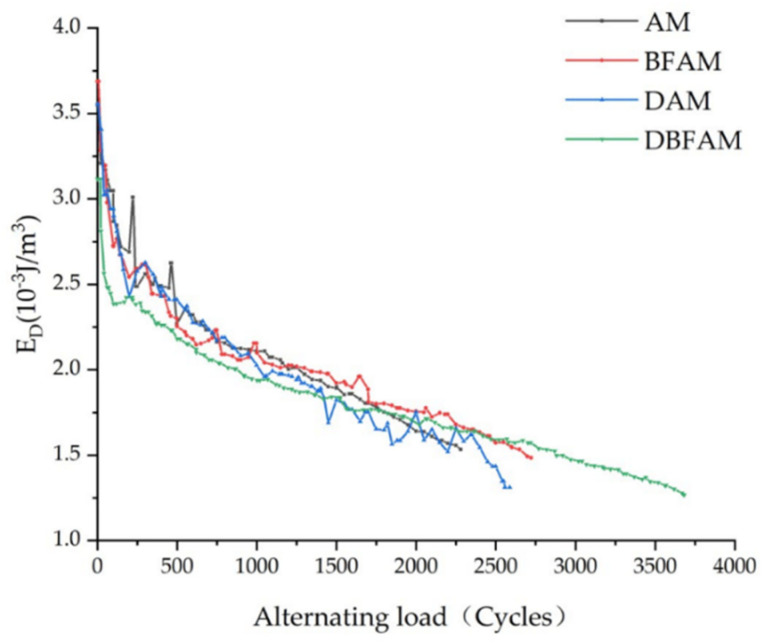
Dissipation energy of asphalt mixture under each loading.

Figure 10 show the changing trend of energy consumption for four types of asphalt mixtures with loading times. It can be seen from the figure that the energy consumption of each loading of four types of asphalt mixtures decreases with the increase of loading times; compared to AM, BFAM and DAM, DBFAM has less dissipation energy per load; therefore, the addition of basalt fiber and diatomite can significantly improve the toughness of asphalt mixture, increase the elastic recovery capacity of asphalt mixture and reduce the dissipated energy under each load.

2. Analysis of cumulative dissipation energy results

The fatigue failure of asphalt mixture is a process of constant energy consumption (*E_CD_*) under an alternating load. According to the theory of fracture mechanics, the fatigue failure of materials is the process of continuous crack propagation, and the energy lost in each loading and unloading cycle is accumulated and transformed into the surface energy of crack propagation to form a new surface. When the accumulated dissipated energy reaches a certain limit during the action of alternating load, the material will undergo fatigue failure.

**Figure 11:**
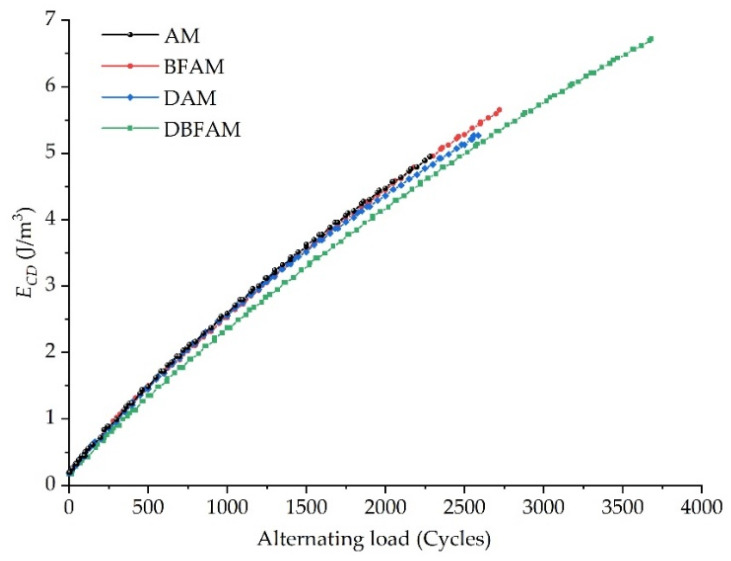
The changing trend of cumulative dissipation energy.

**Figure 12:**
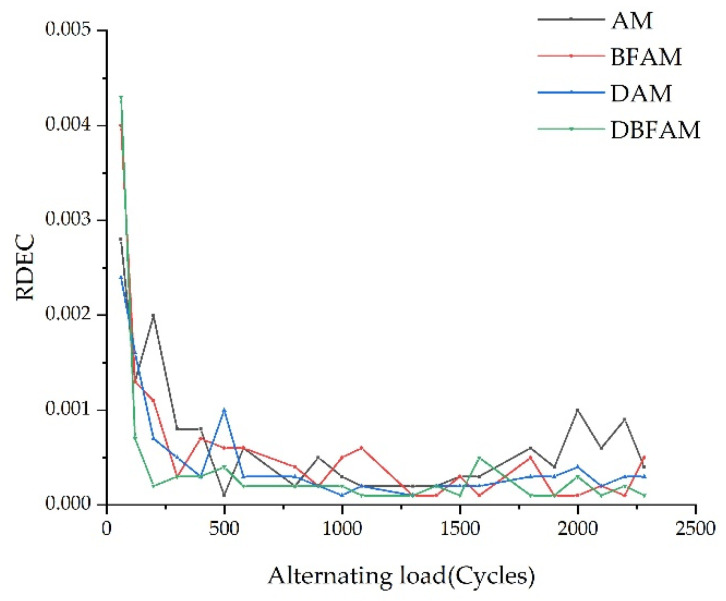
The ratio of dissipated energy change.

The cumulative dissipation energy is calculated according to Equation (6). As shown in Figure 11, the changing trend of cumulative dissipation energy of four types of asphalt mixtures is given. It can be seen from Figure 12 that the cumulative energy consumed by the test piece gradually increases at a certain rate with the increase of the action times of alternating load. The cumulative dissipated energy of basalt fiber modified asphalt mixture and matrix asphalt mixture increases rapidly, followed by diatomite modified asphalt mixture, and the cumulative dissipated energy of composite modified asphalt mixture increases slowly.

Adding basalt fiber and diatomite can improve the elastic recovery ability of asphalt mixture and reduce the energy loss of asphalt mixture caused by work, heat and material damage. The cumulative dissipation energy of DAM is smaller than that of BFAM and AM, which can improve the fatigue performance of the asphalt mixture to a certain extent. Although the cumulative dissipation energy of BFAM is roughly the same as that of AM, the addition of basalt fiber can enhance and prevent cracks. After the addition of fiber, it can prevent the propagation of original defects (microcracks) in the asphalt mixture and effectively delay the initiation of new cracks [14,17,28,44].

3. Analysis of the ratio of dissipated energy change

The variation of the rate of dissipated energy change (*RDEC*) is plotted in Figure 12. It is found that the rate dissipated energy change of asphalt mixture has only two stages, and the third stage does not appear. That is, when the modulus of the asphalt mixture reaches 50%, the failure stage of the asphalt mixture has not been reached [17]. However, *RDEC* in the second stage is in a stable state, so the plateau value (PV) is still calculated by using the average value of the change rate of dissipative energy in the second stage (600–1700 Cycles). The results are shown in Table 9.

It can be seen from Table 9 that the PV value increases slightly after adding basalt fiber. It means that the energy loss of the asphalt mixture could well increase after adding basalt fiber [17,28]. The PV value decreased after adding diatomite and two materials, indicating that the fatigue resistance of DAM and DBFAM is better than AM.

### 3.6. Grey Correlation Analysis of Factors Affecting Fatigue Life

#### 3.6.1. Grey Correlation Analysis Process

When using grey correlation analysis, first select the data column and then analyze the reference number and comparison series in the data. The comparison series and reference series are dimensionless. In order to avoid the different dimensions of each influencing factor, each factor should be dimensionless in the grey correlation analysis; the comparison sequence and reference sequence are engaged in dimensionless processing. Use Equation (8) to calculate the correlation coefficient between the comparison curve and the reference curve at each time and calculate the grey correlation degree. The correlation coefficient is the correlation degree between the comparison sequence and the reference sequence at each time. Therefore, there is more than one correlation coefficient calculated by Equation (8). For analysis, the average value of the calculated correlation coefficient is calculated by Equation (9).

The analysis process of grey correlation degree is as follows:

- The data of the fatigue test and relevant influencing factors are listed in Table 10 to generate the analysis data column; in order to keep the polarity of each influencing factor consistent, the data are processed accordingly. For example, the fatigue performance decreases with the increase of void fraction. In order to keep the polarity consistent, the reciprocal of a void fraction is taken for analysis; the fatigue life decreases with the increase of the initial stiffness modulus. When analyzing the data, take the reciprocal of the initial stiffness modulus for analysis.
- The test data in Table 10 are listed in Table 8 after dimensionless initialization.
- Using the data in Table 11 to calculate the difference sequence, the difference sequence is shown in Table 12.
- According to Table 12, the maximum difference between the two levels is 0.581, and the minimum difference between the two levels is 0. Calculate the grey correlation coefficient according to Equation (8). See Table 13 for the grey correlation coefficient.

#### 3.6.2. Grey Correlation Degree Comparison of Influencing Factors

**Figure 13:**
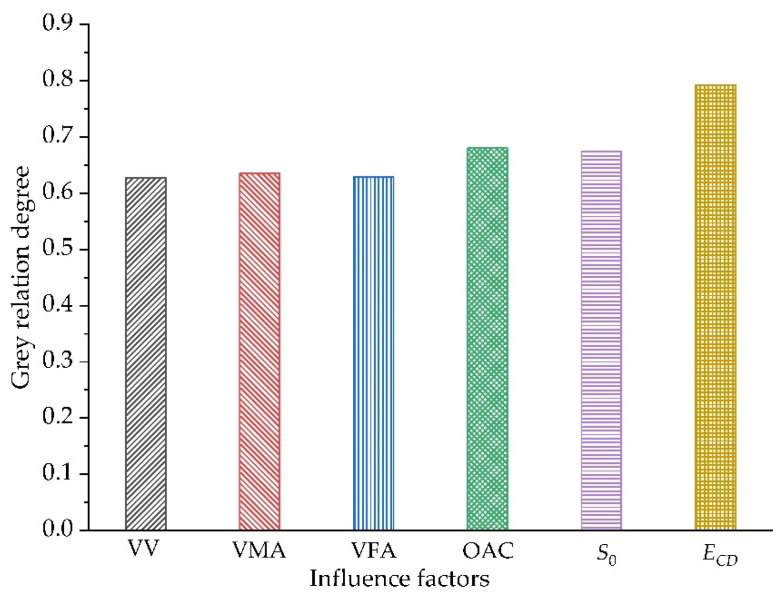
Grey correlation analysis results.

The relation degree between each factor and the fatigue life of the asphalt mixture can be calculated by Equation (9), as shown in Figure 13. From the gray correlation degree ranking in the figure, it can be seen intuitively that the degree of influence is as follows: *E_CD_* > OAC > *S*_0_ > VMA > VFA > VV. Compared with *E_CD_*, OAC, *S*_0_, VMA, VFA and VV have less influence.

In this study, the fatigue test of the asphalt mixture was used to study the effect of diatomite and basalt fiber on the fatigue life of asphalt mixture. The gradation and the specimen forming method of the four types of asphalt mixture are the same. The slight difference in the void ratio is mainly due to the increase of asphalt consent caused by the addition of diatomite and basalt fiber. The addition of basalt fiber and diatomite can affect the fatigue life of the asphalt mixture by affecting the amount of asphalt, the initial stiffness modulus and the cumulated dissipated energy. The mixture of the two has the greatest influence on the cumulative dissipative energy of the asphalt mixture fatigue test, which can greatly improve the fatigue life of asphalt mixture.

## 4. Conclusions

In this study, a Cooper universal pneumatic servo testing machine was used to carry out fatigue tests on four kinds of asphalt mixtures: DBFAM, DAM, BFAM and AM. Through the fatigue test indexes, the influence of basalt fiber and diatomite on the performance of asphalt mixture was analyzed. The effects of OAC, VV, VMA, VFA, initial stiffness modulus and cumulative dissipation energy on the fatigue life of asphalt mixture were analyzed by using the grey correlation theory. The following conclusions can be drawn:

(1) The changing trend in the flexural tensile stiffness modulus of four types of asphalt mixtures is the same as that of flexural tensile stiffness modulus attenuation rate under the alternating load. It can be seen from the flexural tensile stiffness modulus index that the fatigue life of asphalt mixture increases obviously after adding diatomite and basalt fiber, and the improvement effect of adding basalt fiber and diatomite is the best. The fatigue life of DBFAM is 55.7% higher than that of AM.
(2) There is an increasing trend in the lag angle of the four types of asphalt mixture under alternating load, but the increase is slow. The viscoelastic properties of the four types of asphalt mix materials during the fatigue test are constantly changing. With the increase of loading times, the asphalt mixture continues to develop in the direction of viscosity.
(3) The energy consumption of each loading decreases with the increase of loading times. Compared with AM, the energy consumption of DAM, BFAM, DBFAM is small under each loading. The cumulative dissipation energy and the plateau value of DBFAM are the smallest. The addition of diatomite and basalt fiber can significantly improve the toughness of asphalt mixture so that it increases the elastic recovery capacity of the asphalt mixture. When basalt fiber and diatomite are added at the same time, the improvement effect of elastic recovery ability of asphalt mixture is the most obvious.
(4) Through the grey correlation analysis, it can be seen that the cumulative dissipation energy, initial stiffness modulus and OAC have a great impact on the fatigue life of asphalt mixture, and the cumulative dissipation energy is the most significant. After adding diatomite and basalt fiber, the cumulative dissipation energy is greatly improved, and then the fatigue life of the asphalt mixture is increased.

## Figures and Tables

**Table 1:** Physical properties of AH-90 asphalt.

Properties	Values	Standard Values
Density (15 °C, g/cm^3^)	1.016	—
Penetration (25 °C, 0.1 mm)	91.8	80–100
Softening point T_R&B_ (°C)	46.9	≥45
Ductility (25 °C, cm)	>150	≥100
Viscosity (135 °C, Pa·s)	306.9	—
After TFOT		
Mass loss (%)	0.38	≤+0.8
Residual penetration ratio (25 °C, %)	73.3	≥57
Softening point T_R&B_ (°C)	49.6	—
Ductility (15 °C, cm)	>120	≥20
Viscosity (135 °C, Pa·s)	432.5	—

**Table 2:** Properties of diatomite.

Properties	Color	pH	Specific Gravity (g/cm^3^)	Bulk Density (g/cm^3^)
Value	Off-white	7–8	2.0–2.2	0.34–0.41

**Table 3:** The mineral composition of diatomite (provided by manufacturer).

Mineral Composition	SiO_2_	Al_2_O_3_	Fe_2_O_3_	CaO	MgO	TiO_2_	K_2_O	Loss on Ignition
Content (%)	85.60	4.50	1.50	0.52	0.45	0.30	0.67	4.61

**Table 4:** The mineral composition of basalt fiber (provided by manufacturer).

Mineral Composition	SiO_2_	Al_2_O_3_	Fe_2_O_3_	CaO	MgO	Ti_2_O	Na_2_O	Others
Content (%)	50	15	12	10	5	1.5	4	2.5

**Table 5:** Properties of basalt fiber (provided by manufacturer).

Properties	Values	Standard Values
Diameter (μm)	10–13	—
Length (mm)	6	—
Water content (%)	0.030	≤0.2
Combustible content (%)	0.56	—
Linear density (Tex)	2398	2400 ± 120
Breaking strength (N/Tex)	0.55	≥0.40
Tensile strength (MPa)	2320	≥2000
Tensile modulus of elasticity (GPa)	86.3	≥85
Elongation at break (%)	2.84	≥2.5

**Table 6:** Aggregate test results and specifications index.

Properties	Value	
Diameter mm	13.2~16	9.5~13.2
Bulk specific gravity (g/cm^3^)	2.810	2.805
Gross volume relative density (g/cm^3^)	2.784	2.771
Water absorption (%)	0.33	0.44

**Table 7:** Coarse aggregate test results and specifications index.

Properties	Value	Standard Value
Crushing value (%)	8.9	≤26
Percentage of flat-elongated particles (≥9.5 mm) (%)	3.6	≤8
Percentage of flat-elongated particles (<9.5 mm) (%)	4.8	≤12
Abrasion value (%)	12	≤28

**Table 8:** Physical properties of asphalt mixtures.

Indicators	AM	BFAM	DAM	DBFAM
OAC (%)	4.78	5.09	5.12	5.22
VV (%)	4.098	4.065	4.065	4.20
VMA (%)	15.6	16.2	15.4	16
VFA (%)	73.7	74.9	73.6	73.8

**Table 9:** The Plateau Value.

Asphalt Mixture Type	Strain Level (με)	PV
AM	800	2.41 × 10^−4^
BFAM	800	2.47 × 10^−4^
DAM	800	2.11 × 10^−4^
DBFAM	800	1.96 × 10^−4^

**Table 10:** Raw data.

Influence Factor	AM	BFAM	DAM	DBFAM
OAC	4.78	5.09	5.12	5.22
1/VV	0.244	0.246	0.246	0.238
VMA	15.6	16.2	15.4	16
VFA	73.7	74.9	73.6	73.8
1/*S*_0_	0.000137	0.00014	0.000146	0.00016
*E_CD_*	4.635	5.375	5.212	6.154
Fatigue life	2099	2549	2541	3269

**Table 11:** Data initialization results.

Influence Factor	AM	BFAM	DAM	DBFAM
OAC	1.000	1.065	1.071	1.092
1/VV	1.000	1.007	1.010	0.976
VMA	1.000	1.038	0.987	1.026
VFA	1.000	1.016	0.999	1.001
1/*S*_0_	1.000	1.027	1.065	1.163
*E_CD_*	1.000	1.160	1.124	1.328
Fatigue life	1.000	1.214	1.211	1.557

**Table 12:** Difference sequence.

Influence Factor	AM	BFAM	DAM	DBFAM
OAC	0.000	0.150	0.139	0.465
1/VV	0.000	0.207	0.201	0.581
VMA	0.000	0.176	0.223	0.532
VFA	0.000	0.198	0.212	0.556
1/*S*_0_	0.000	0.187	0.146	0.394
*E_CD_*	0.000	0.055	0.086	0.230

**Table 13:** Grey correlation coefficient results.

Influence Factor	AM	BFAM	DAM	DBFAM
OAC	1.000	0.660	0.676	0.384
1/VV	1.000	0.584	0.591	0.333
VMA	1.000	0.623	0.565	0.353
VFA	1.000	0.595	0.578	0.343
1/*S*_0_	1.000	0.608	0.666	0.424
*E_CD_*	1.000	0.842	0.771	0.559

## Data Availability

The testing and analysis data used to support the findings of this study are included in the article.
